# Prospective Phase II Trial of Biweekly Gemcitabine Plus Nab‐Paclitaxel as First‐Line Therapy in Patients Aged 75 Years and Older With Unresectable Pancreatic Cancer

**DOI:** 10.1002/cnr2.70691

**Published:** 2026-09-14

**Authors:** Kenji Ikezawa, Toshihiro Imai, Ryoji Takada, Takuo Yamai, Nobuyasu Fukutake, Makiko Urabe, Yugo Kai, Kaori Mukai, Tasuku Nakabori, Kazuyoshi Ohkawa

**Affiliations:** ^1^ Department of Hepatobiliary and Pancreatic Oncology Osaka International Cancer Institute Osaka Japan; ^2^ Department of Gastroenterology and Hepatology Kansai Rosai Hospital Amagasaki Japan; ^3^ Department of Gastroenterology and Hepatology Itami City Hospital Itami Japan; ^4^ Department of Gastroenterology Osaka General Medical Center Osaka Japan; ^5^ Department of Gastroenterology and Hepatology Ikeda Municipal Hospital Ikeda Japan

**Keywords:** chemotherapy, geriatric oncology, modified schedule, older adults, time toxicity

## Abstract

**Background:**

Although older adults (≥ 75 years) constitute a large proportion of patients with unresectable pancreatic cancer (PC), they remain underrepresented in clinical trials. Biweekly gemcitabine plus nab‐paclitaxel (GnP) has been evaluated in retrospective studies as an alternative dosing schedule, whereas prospective evidence remains limited.

**Aims:**

This single‐center, prospective Phase II trial aimed to evaluate the efficacy and safety of biweekly GnP as a first‐line therapy in older adults with unresectable PC.

**Methods and Results:**

Eligible patients aged ≥ 75 years with histologically or cytologically confirmed unresectable pancreatic adenocarcinoma received GnP (1000 mg/m^2^ gemcitabine; 125 mg/m^2^ nab‐paclitaxel) on days 1 and 15 of each 28‐day cycle. The primary endpoint was the overall response rate (ORR). Key secondary endpoints included overall survival (OS), progression‐free survival (PFS), and safety.

A total of 17 patients were enrolled between August 2019 and March 2021 (median age, 77 years; 13 metastatic, 4 locally advanced). The ORR was 47.1% (8/17; 95% confidence interval [CI], 23.0%–72.2%), and the disease control rate was 82.4% (14/17). The median OS and PFS were 16.8 (95% CI, 5.9–24.6) and 8.3 (95% CI, 4.9–10.1) months, respectively. The 1‐ and 2‐year survival rates were 58.8% and 29.4%, respectively. Grade ≥ 3 hematologic adverse events occurred in 17.6% of patients. Treatment‐related adverse events led to treatment discontinuation in three patients: one case of interstitial pneumonia and two cases of peripheral sensory neuropathy. No treatment‐related deaths occurred.

**Conclusion:**

Biweekly GnP showed promising antitumor activity in a selected population of patients aged ≥ 75 years with unresectable PC. Because enrollment ended before the planned sample size was reached, the study findings should be interpreted cautiously.

**Trial Registration:** jRCTs051190038

## 1 Introduction

Pancreatic cancer (PC) is one of the most lethal malignancies worldwide and the third leading cause of cancer‐related mortality in the United States and Japan [1, 2]. Its incidence is continuously rising, primarily affecting older adults [1, 2, 3]. For example, in the United States, approximately 37% of patients newly diagnosed with PC are aged ≥ 75 years [4]. Current treatment guidelines for unresectable PC support the use of systemic chemotherapy based on the pivotal MPACT (gemcitabine plus nab‐paclitaxel [GnP]), ACCORD‐11 (fluorouracil, leucovorin, irinotecan, plus oxaliplatin [FOLFIRINOX]), and NAPOLI‐3 (liposomal irinotecan, oxaliplatin, leucovorin, plus fluorouracil [NALIRIFOX]) trials, as well as subsequent studies [5, 6, 7, 8, 9, 10, 11]. Among these therapeutic approaches, GnP is established as the first‐line regimen for metastatic and locally advanced PC, supported by the results of the MPACT trial and other studies [9, 12, 13]. However, older adults remain underrepresented in pivotal trials and are more susceptible to chemotherapy‐related toxicities, leading to frequent dose reductions or discontinuations [14, 15, 16]. Although retrospective studies demonstrate favorable outcomes in older patients receiving standard GnP, the dose intensity for this population is substantially reduced compared with that for younger patients. A multicenter retrospective study indicated a median overall survival (OS) of 21.8 months for patients with locally advanced disease and 12.1 months for those with metastatic disease among individuals aged ≥ 75 years [17]. Ishimoto et al. observed similar efficacy of GnP in older and younger cohorts, despite decreased dose intensity in the older cohort [18].

To minimize adverse effects while maintaining treatment effectiveness, several studies have investigated alternative dosing schedules of GnP. For example, in a multicenter Phase I/II trial in patients with PC and Eastern Cooperative Oncology Group (ECOG) performance status (PS) 2, Macarulla et al. assessed modified GnP dosing schedules and demonstrated that these regimens were feasible with acceptable safety and efficacy [19]. In a retrospective study of metastatic PC, Ahn et al. investigated a biweekly GnP regimen, in which both agents were administered on Days 1 and 15 of a 28‐day cycle [20]. These authors reported a median OS of 10 months and a median progression‐free survival (PFS) of 5.4 months, with a generally favorable safety profile. Similarly, in a retrospective study utilizing a prospectively collected database, Kokkali et al. reported outcomes for 46 patients with advanced PC who received a biweekly GnP regimen at the similar dose intensity as the conventional schedule [21]. Notably, the rates of adverse events (AEs) were comparable to those of the original regimen, with median OS and PFS of 10 and 5 months, respectively. A biweekly GnP regimen has also been evaluated specifically in older patients with advanced PC [22]. However, prospective evidence for biweekly GnP in older adults with unresectable PC remains limited [23].

Therefore, in this single‐center, prospective Phase II trial, we aimed to assess the efficacy and safety of biweekly GnP as first‐line therapy in patients aged ≥ 75 years with unresectable PC.

## 2 Methods

### 2.1 Study Design and Patients

This single‐center, open‐label, prospective Phase II trial was conducted at the Osaka International Cancer Institute, Japan (jRCTs051190038). The study protocol adhered to the ethical guidelines of the Declaration of Helsinki and Clinical Trials Act in Japan and was approved by the Institutional Review Board of the Osaka International Cancer Institute (approval No. 19076). Written informed consent was obtained from all participants prior to enrollment.

Eligible patients were aged ≥ 75 years and had histologically or cytologically confirmed adenocarcinoma, with imaging findings consistent with PC. Unresectable disease was diagnosed based on chest, abdominal, and pelvic imaging, confirming either unresectable locally advanced or metastatic PC, based on the National Comprehensive Cancer Network (NCCN) guidelines [24]. Patients were also required to have no uncontrollable pleural or peritoneal effusion, an ECOG PS of 0 or 1, and a life expectancy of ≥ 3 months at enrollment. Patients with a history of chemotherapy or surgical resection for PC were excluded, except for those who had undergone laparotomy alone or palliative gastrointestinal bypass surgery. Patients with a history of chemotherapy or radiotherapy for other malignancies were eligible only if at least 6 months had passed since treatment completion. Adequate organ function was also required and defined as a white blood cell count of 3000–12 000/μL, absolute neutrophil count ≥ 1500/μL, hemoglobin ≥ 9.0 g/dL, platelet count ≥ 100 000/μL, serum albumin ≥ 3.0 g/dL, total bilirubin ≤ 2.0 mg/dL, aspartate aminotransferase and alanine aminotransferase ≤ 3.0 × the upper limit of normal (ULN) (or ≤ 5 × ULN in patients with liver metastases), and serum creatinine ≤ 1.2 mg/dL or creatinine clearance ≥ 50 mL/min.

Key exclusion criteria included active infection requiring systemic therapy, including human immunodeficiency virus infection, active tuberculosis, or uncontrolled bacterial infection, but excluding well‐controlled viral hepatitis or inactive pulmonary tuberculosis; uncontrolled comorbidities, such as severe cardiac disease, renal failure, hepatic failure, intestinal obstruction, paralytic ileus, or poorly controlled diabetes mellitus (HbA1c ≥ 10%); unstable angina, with onset within the past 3 weeks or worsening in frequency/severity; a history of myocardial or cerebral infarction within the past 6 months; and interstitial lung disease.

Patient data collected at enrollment included ECOG PS, body mass index, past medical history, Geriatric‐8 (G8) score (an eight‐item geriatric screening tool; range, 0–17; lower scores indicate greater geriatric vulnerability) [25], clinical stage, and laboratory results.

### 2.2 Treatment Protocol

Patients received gemcitabine 1000 mg/m^2^ and nab‐paclitaxel 125 mg/m^2^ intravenously on Days 1 and 15 of a 28‐day cycle. Nab‐paclitaxel was infused over 30 min, followed by gemcitabine over 30 min. Protocol treatment continued until a discontinuation criterion was met, including disease progression, an AE that prevented continuation of protocol treatment, a patient request to discontinue treatment, death during treatment, or other circumstances making a continuation of protocol treatment inappropriate.

For Day 1 of Cycle 2 and subsequent cycles and for Day 15 dosing, treatment required an absolute neutrophil count ≥ 1000/μL, platelet count ≥ 75 000/μL, total bilirubin concentration ≤ 3.0 mg/dL, aspartate aminotransferase and alanine aminotransferase levels ≤ 100 U/L, and acceptable levels of nonhematologic toxicity. If any criterion for Day 1 of Cycle 2 or a subsequent cycle was not met, the start of the cycle was delayed. If any criterion for Day 15 treatment was not met, the Day 15 dose was skipped. When Day 15 was skipped, Day 22 became the planned Day 1 of the next cycle, and both drugs were restarted one dose level lower. Nab‐paclitaxel could be administered only if peripheral neuropathy was Grade 0–2 without substantial interference with daily activities; for Grade 2 neuropathy, a one‐level reduction of nab‐paclitaxel could be considered at the investigator's discretion.

Dose levels were prespecified as gemcitabine 1000, 800, and 600 mg/m^2^ and nab‐paclitaxel 125, 100, and 75 mg/m^2^. Dose reductions were made according to protocol‐defined criteria for hematologic toxicities, including a neutrophil count < 500/μL, platelet count < 50 000/μL, or febrile neutropenia, as well as for nonhematologic toxicities. Once the dose of a drug had been reduced, re‐escalation of that drug dose was not permitted.

### 2.3 Assessments

Tumor response was evaluated by the investigators every 8 weeks based on contrast‐enhanced computed tomography following the Response Evaluation Criteria in Solid Tumors (RECIST) version 1.1. Tumor marker levels were measured every 4 weeks. AEs were defined as any unfavorable or unintended disease or sign, including laboratory abnormalities, occurring in a study participant regardless of its causal relationship to the study treatment. A serious AE (SAE) was defined as an AE that resulted in death, was life‐threatening, required hospitalization for treatment, prolonged hospitalization, resulted in persistent or substantial disability, caused a congenital anomaly in offspring, or was otherwise considered medically important. AEs were recorded and graded using the Common Terminology Criteria for Adverse Events (CTCAE) version 5.0. Blood tests and toxicities were assessed on Days 1 and 15 of each cycle. Follow‐up assessments, including subsequent anticancer therapy and survival status, continued until death or the last date of confirmed survival, with a follow‐up period of 2 years after the enrollment of the last patient.

### 2.4 Endpoints

The primary endpoint was the overall response rate (ORR), defined as the proportion of eligible patients with measurable disease achieving complete or partial response as their best overall response per RECIST version 1.1. Secondary endpoints included the disease control rate, OS, PFS, 1‐ and 2‐year survival rates, relative dose intensity of each study drug, and incidence of AEs. OS was defined as the time from enrollment to death from any cause; patients alive were censored at the last date of confirmed survival. PFS was defined as the time from enrollment to disease progression or death from any cause, whichever occurred first.

### 2.5 Statistical Considerations

This study employed Simon's two‐stage Minimax design. As no prospective data on the response rate of biweekly GnP were available, ORRs of 23% from the MPACT trial and 58% from a domestic Phase II trial for standard GnP, as well as 7% for gemcitabine monotherapy, were used as references [9, 26]. Based on these data, the expected ORR for this study was set at 30% and the threshold ORR at 7%. With a one‐sided alpha of 0.05 and 80% power, the required sample size was 18 patients. In Stage 1, seven evaluable patients were assessed; continuation to Stage 2 was considered if at least one response was observed (ORR ≥ 14.3%), together with a review of SAEs and the appropriateness of study continuation. To account for an anticipated dropout rate of approximately 10%, the planned enrollment target was 20 patients. Although the planned sample size was 20, patient accrual was terminated at 17 owing to an unstable supply of nab‐paclitaxel during the study period [27, 28].

The 95% confidence interval (CI) for the ORR was calculated using the exact binomial method. OS and PFS were estimated using the Kaplan–Meier method with two‐sided 95% CIs. All statistical analyses were performed using EZR, a graphical interface of the R Commander software package for Windows (version 1.54) (Saitama Medical Center, Jichi Medical University, Saitama, Japan) [29].

## 3 Results

### 3.1 Patient Enrollment and Baseline Characteristics

Between August 2019 and March 2021, 17 patients were enrolled in this study, and the baseline patient characteristics are summarized in Table 1. The median age was 77 (range, 75–81) years, and five patients (29.4%) were male. A total of 11 patients (64.7%) had ECOG PS 0, and six patients (35.3%) had ECOG PS 1. The median body mass index was 23.4 (range, 17.4–34.5) kg/m^2^. Most patients had metastatic disease (13/17, 76.5%), whereas four (23.5%) had locally advanced disease. All 17 enrolled patients had at least one measurable lesion at baseline according to RECIST version 1.1 and were included in the ORR analysis. The median G8 score was 13 (range, 6–17). Three patients (17.6%) had undergone biliary drainage. Many patients had comorbidities, including hypertension (12 patients), hyperlipidemia (7 patients), diabetes mellitus (6 patients), and hyperuricemia (1 patient). The median serum carbohydrate antigen 19–9 and albumin levels were 950 (range, 2–39 164) U/mL and 3.8 (range, 3.1–4.3) g/dL, respectively.

**TABLE 1:** Patient characteristics.

		Total (*N* = 17)	Metastatic (*n* = 13)	Locally advanced (*n* = 4)
Age (years)	Median (range)	77 (75–81)	76 (75–81)	79 (75–81)
Sex	Male, *n* (%)	5 (29.4)	5 (38.5)	0 (0)
	Female, *n* (%)	12 (70.6)	8 (61.5)	4 (100)
Body mass index (kg/m^2^)	Median (range)	23.4 (17.4–34.5)	23.4 (17.4–34.5)	22.9 (18.0–26.7)
Location of pancreatic tumor	Head, *n* (%)	7 (41.2)	4 (30.8)	3 (75.0)
	Body/tail, *n* (%)	10 (58.8)	9 (69.2)	1 (25.0)
Metastatic site^a^	Liver	4	4	—
	Lymph nodes	7	7	—
	Lung	6	6	—
	Peritoneum	3	3	—
	Adrenal gland	1	1	—
Number of metastatic sites	1	7	7	—
	2	4	4	—
	3	2	2	—
ECOG PS	0, *n* (%)	11 (64.7)	11 (84.6)	0 (0)
	1, *n* (%)	6 (35.3)	2 (15.4)	4 (100)
Geriatric‐8 score	Median (range)	13 (6–17)	13 (8–17)	11 (6–14)
Smoking history	Current smoker, *n* (%)	2 (11.8)	2 (15.4)	0 (0)
	Past smoker, *n* (%)	3 (17.6)	3 (23.1)	0 (0)
	Never smoker, *n* (%)	12 (70.6)	8 (61.5)	4 (100)
Biliary drainage	Yes, *n* (%)	3 (17.6)	1 (7.7)	2 (50.0)
	No, *n* (%)	14 (82.4)	12 (92.3)	2 (50.0)
White blood cell count (/μL)	Median (range)	5610 (3000–8000)	5610 (3000–8000)	5525 (4140–7900)
Hemoglobin (g/dL)	Median (range)	12.3 (10.7–14.2)	12.7 (10.7–14.2)	11.8 (10.9–13.2)
Platelet count (×10^4^/μL)	Median (range)	21.2 (10.8–42.1)	18.1 (10.8–30.0)	25.0 (20.9–42.1)
Neutrophil count (/μL)	Median (range)	3860 (1850–5930)	3860 (1850–5860)	3655 (2610–5930)
Total bilirubin (mg/dL)	Median (range)	0.7 (0.4–1.0)	0.8 (0.4–1.0)	0.6 (0.4–1.0)
AST (U/L)	Median (range)	19 (14–34)	21 (17–34)	16 (14–18)
ALT (U/L)	Median (range)	17 (10–38)	17 (11–38)	16 (10–25)
Creatinine (mg/dL)	Median (range)	0.58 (0.33–0.82)	0.65 (0.33–0.82)	0.49 (0.45–0.55)
Albumin (g/dL)	Median (range)	3.8 (3.1–4.3)	3.8 (3.1–4.3)	3.6 (3.1–4.0)
CRP (mg/dL)	Median (range)	0.12 (0.01–5.00)	0.12 (0.02–5.00)	0.22 (0.01–0.50)
HbA1c (%)	Median (range)	6.3 (5.6–9.9)	6.9 (5.6–9.9)	5.9 (5.6–6.6)
Serum CEA level (ng/mL)	Median (range)	4.2 (1.1–246.3)	16.7 (1.1–246.3)	2.9 (1.4–3.6)
Serum CA19‐9 level (U/mL)	Median (range)	950 (2–39 164)	2259 (2–39 164)	496 (12–1138)
Serum DUPAN‐2 level (U/mL)	Median (range)	173 (25–4800)	301 (25–4800)	54 (39–296)

Abbreviations: ALT, alanine aminotransferase; AST, aspartate aminotransferase; CA19‐9, carbohydrate antigen 19‐9; CEA, carcinoembryonic antigen; CRP, C‐reactive protein; DUPAN‐2, Duke pancreatic monoclonal antigen Type 2; ECOG PS, Eastern Cooperative Oncology Group performance status.

^a^ Metastatic sites were not mutually exclusive; patients may have more than one metastatic site.

### 3.2 Treatment Delivery

**FIGURE 1:**
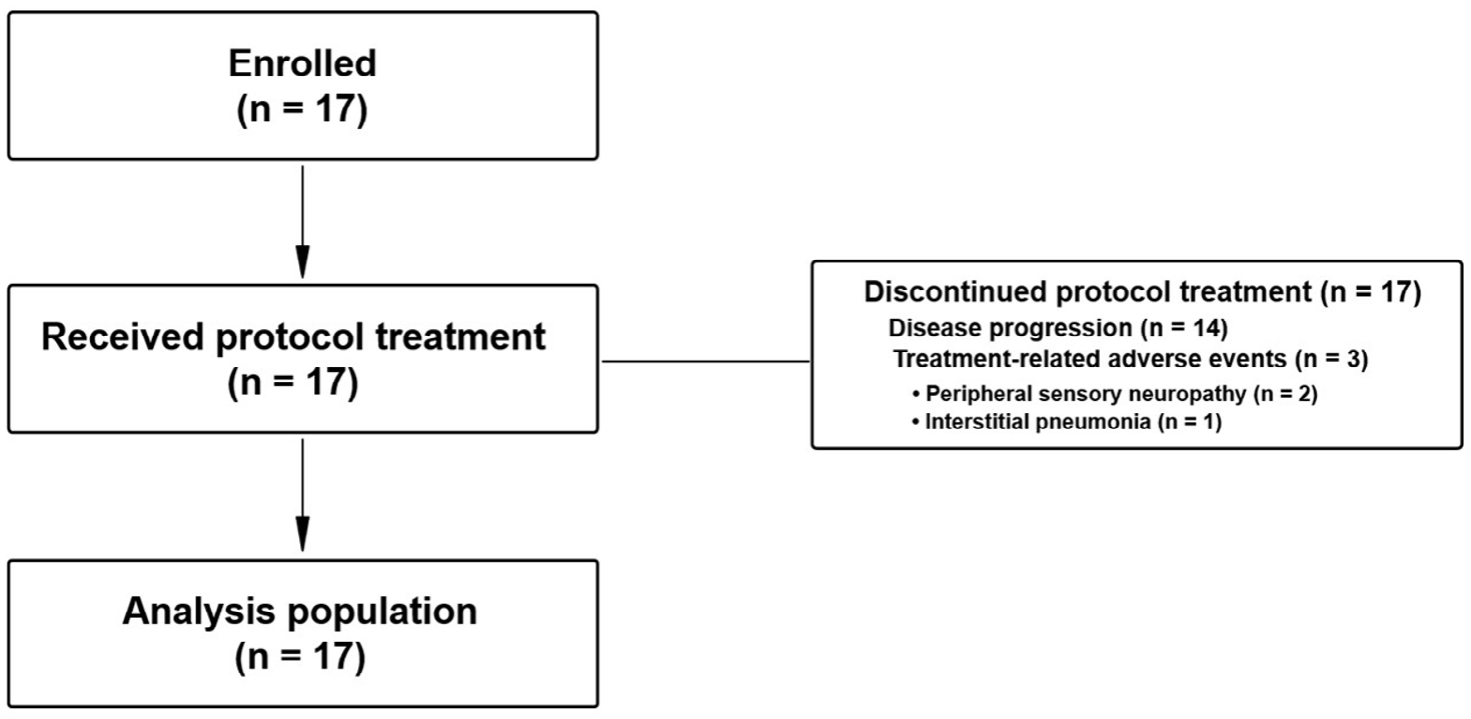
Patient flow diagram.

The median duration of treatment was 5.5 (range, 1.8–15.2) months, with a median of 6.0 (range, 2.0–16.5) cycles (Table 2). The median relative dose intensity was 100% (range, 63.0%–100.0%) for gemcitabine and 87.3% (range, 56.8%–100.0%) for nab‐paclitaxel. At least one treatment delay occurred in five patients (29.4%). Dose reduction occurred in 11 patients (64.7%): six (35.3%) for gemcitabine and 11 (64.7%) for nab‐paclitaxel. All 17 patients discontinued protocol treatment: 14 because of disease progression and three because of treatment‐related AEs (peripheral sensory neuropathy, *n* = 2; interstitial pneumonia, *n* = 1) (Figure 1).

**TABLE 2:** Study treatment administration.

Parameter	Total (*N* = 17)	Metastatic (*n* = 13)	Locally advanced (*n* = 4)
Overall			
Duration of treatment (months), median (range)	5.5 (1.8–15.2)	5.5 (1.8–15.2)	5.3 (1.8–11.8)
Number of cycles, median (range)	6.0 (2.0–16.5)	6.0 (2.0–16.5)	5.5 (2.0–12.0)
Delay in ≥ 1 cycle, *n* (%)	5 (29.4)	3 (23.1)	2 (50.0)
Dose reduction in ≥ 1 cycle, *n* (%)	11 (64.7)	9 (69.2)	2 (50.0)
Gemcitabine			
Dose reduction in ≥ 1 cycle, *n* (%)	6 (35.3)	4 (30.8)	2 (50.0)
Relative dose intensity, % (range)	100 (63.0–100)	100 (63.0–100)	91.9 (72.3–100)
Nab‐paclitaxel			
Dose reduction in ≥ 1 cycle, *n* (%)	11 (64.7)	9 (69.2)	2 (50.0)
Relative dose intensity, % (range)	87.3 (56.8–100)	87.3 (56.8–100)	91.9 (72.3–100)

### 3.3 Efficacy

**FIGURE 2:**
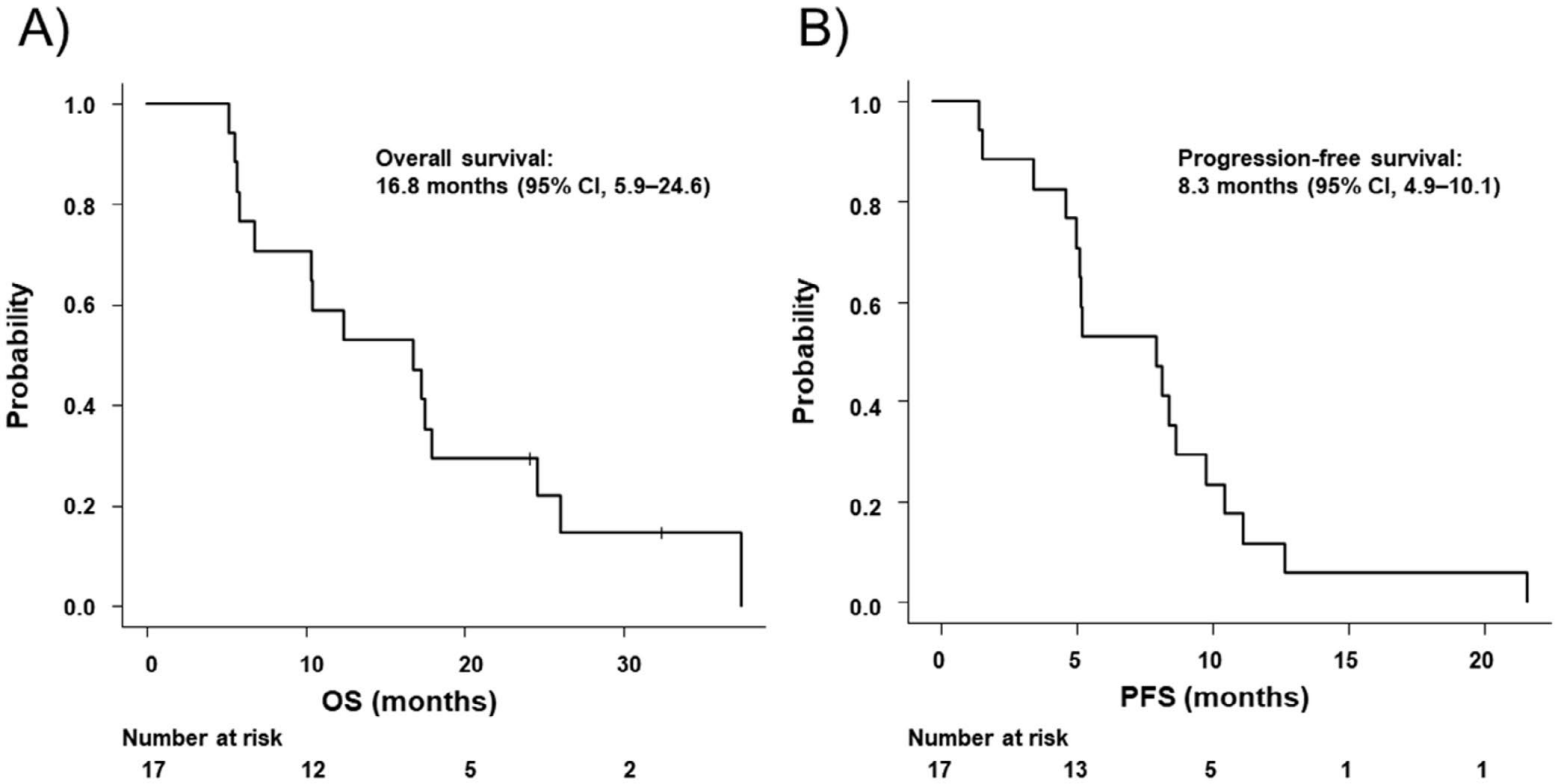
Kaplan–Meier curves for overall and progression‐free survival. (A) Overall survival. (B) Progression‐free survival. CI, confidence interval; OS, overall survival; PFS, progression‐free survival.

The prespecified Stage 1 continuation criterion was met, and the study proceeded to Stage 2. At data cutoff (March 31, 2023), the median follow‐up was 16.8 (range, 5.2–37.4) months, and two patients were alive. The confirmed ORR was 47.1% (8/17; 95% CI, 23.0%–72.2%; all partial responses), and the disease control rate was 82.4% (14/17) (Table 3). The median OS and PFS were 16.8 (95% CI, 5.9–24.6) and 8.3 (95% CI, 4.9–10.1) months, respectively (Figure 2). The 1‐ and 2‐year survival rates were 58.8% (10/17) and 29.4% (5/17), respectively. Fourteen patients (14/17, 82.4%) received subsequent treatment: S‐1 (oral fluoropyrimidine; *n* = 7), 5‐fluorouracil/levoleucovorin plus nanoliposomal irinotecan (*n* = 4), gemcitabine monotherapy (*n* = 2), and gemcitabine with radiotherapy (*n* = 1). Among the two patients who subsequently received gemcitabine monotherapy, one continued gemcitabine after discontinuation of nab‐paclitaxel because of toxicity, whereas the other received reduced‐dose gemcitabine monotherapy after disease progression because the patient declined a switch to another systemic regimen.

**TABLE 3:** Best‐confirmed tumor response.

Best‐confirmed overall tumor response, *n* (%)	Total (*N* = 17)	Metastatic (*n* = 13)	Locally advanced (*n* = 4)
Complete response	0 (0)	0 (0)	0 (0)
Partial response	8 (47.1)	7 (53.8)	1 (25.0)
Stable disease	6 (35.3)	4 (30.8)	2 (50.0)
Progressive disease	3 (17.6)	2 (15.4)	1 (25.0)
Not evaluated	0 (0)	0 (0)	0 (0)
Overall response rate, *n* (%)	8 (47.1)	7 (53.8)	1 (25.0)
95% confidence interval for overall response rate, %	23.0–72.2	25.1–80.8	0.6–80.6
Disease control rate, *n* (%)	14 (82.4)	11 (84.6)	3 (75.0)

**FIGURE 3:**
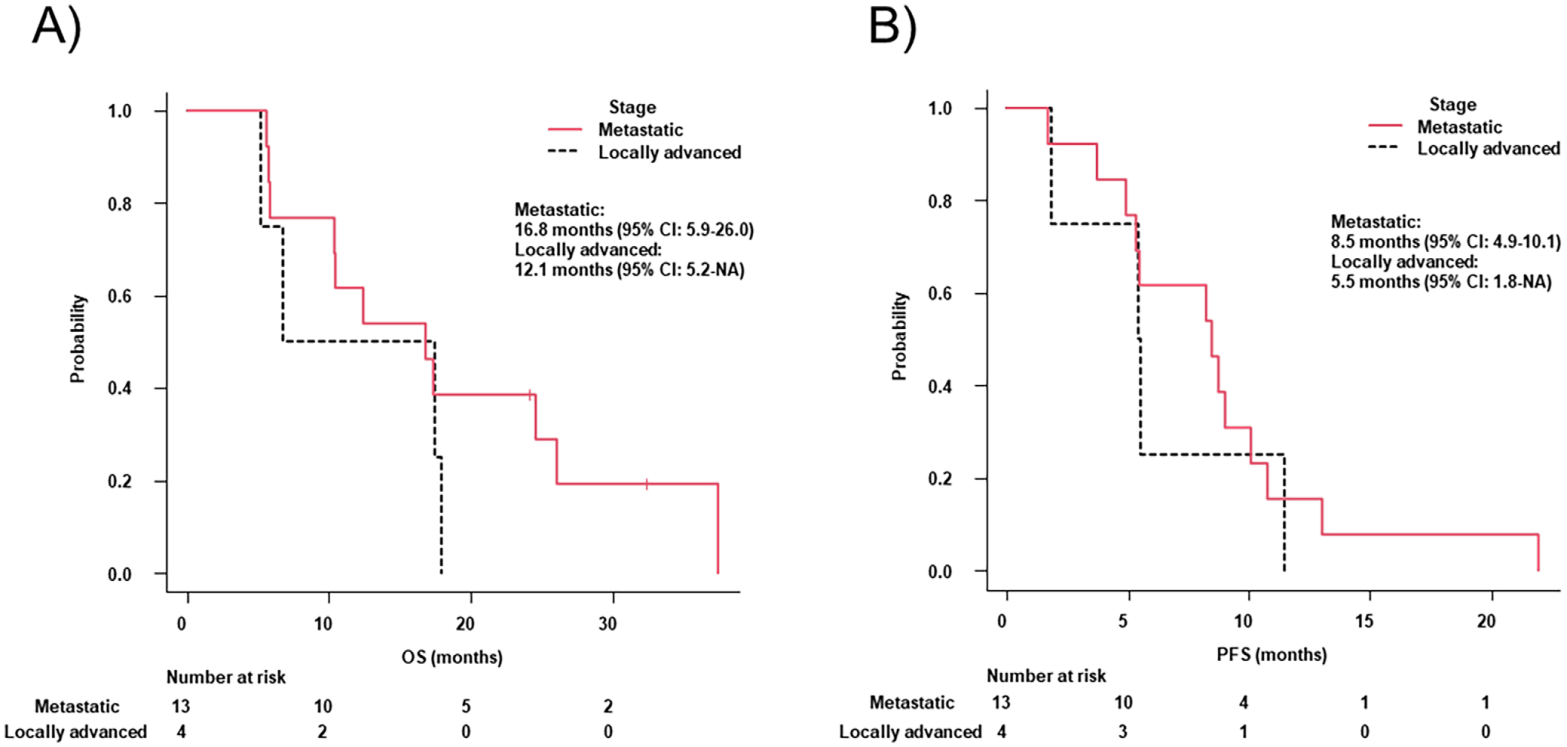
Kaplan–Meier curves for overall and progression‐free survival stratified by disease extent. (A) Overall survival in patients with metastatic disease and those with locally advanced disease. (B) Progression‐free survival in patients with metastatic disease and those with locally advanced disease. CI, confidence interval; NA, not available; OS, overall survival; PFS, progression‐free survival.

**FIGURE 4:**
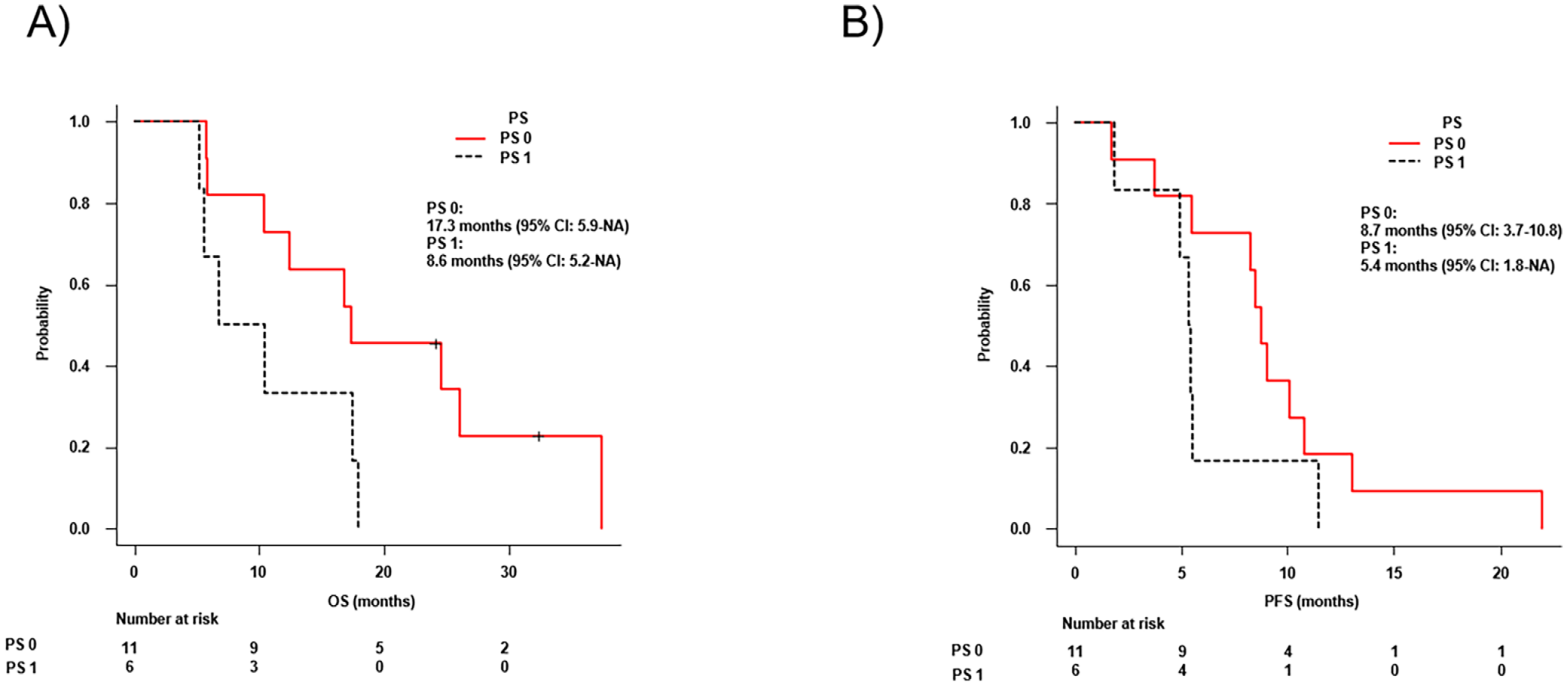
Kaplan–Meier curves for overall and progression‐free survival stratified by ECOG performance status. (A) Overall survival in patients with ECOG PS 0 and those with ECOG PS 1. (B) Progression‐free survival in patients with ECOG PS 0 and those with ECOG PS 1. CI, confidence interval; ECOG, Eastern Cooperative Oncology Group; NA, not available; OS, overall survival; PFS, progression‐free survival; PS, performance status.

**FIGURE 5:**
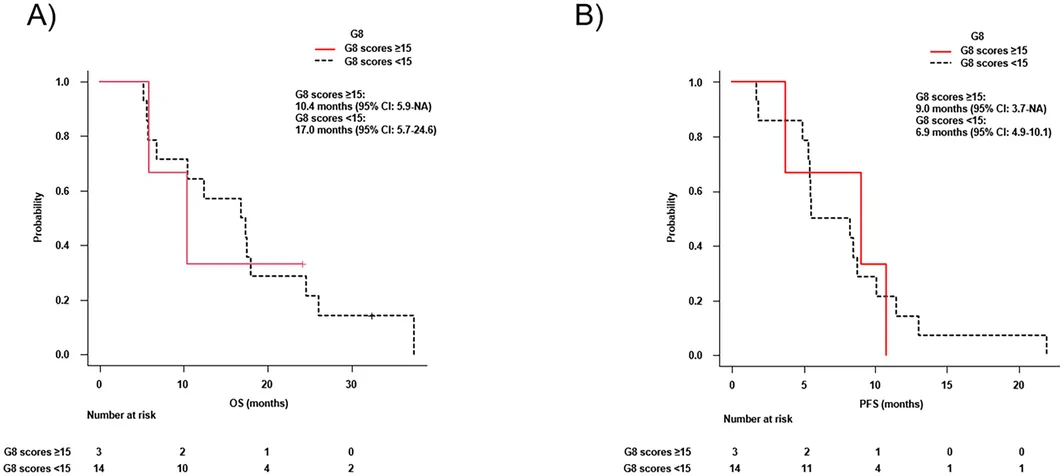
Kaplan–Meier curves for overall and progression‐free survival stratified by Geriatric‐8 score. (A) Overall survival in patients with G8 scores ≥ 15 and those with G8 scores < 15. (B) Progression‐free survival in patients with G8 scores ≥ 15 and those with G8 scores < 15. CI, confidence interval; G8, Geriatric‐8; NA, not available; OS, overall survival; PFS, progression‐free survival.

We performed exploratory survival analyses stratified by disease stage, ECOG PS, and G8 score. Median OS was 16.8 (95% CI, 5.9–26.0) months in patients with metastatic disease (*n* = 13) and 12.1 (95% CI, 5.2–not available [NA]) months in those with locally advanced disease (*n* = 4), and median PFS was 8.5 (95% CI, 4.9–10.1) months and 5.5 (95% CI, 1.8–NA) months, respectively (Figure 3). When stratified by PS, median OS was 17.3 (95% CI, 5.9–NA) months in patients with ECOG PS 0 (*n* = 11) compared with 8.6 (95% CI, 5.2–NA) months in those with ECOG PS 1 (*n* = 6) (Figure 4). Median PFS was 8.7 (95% CI, 3.7–10.8) months and 5.4 (95% CI, 1.8–NA) months, respectively. Using the G8 cutoff of 15, median OS was 10.4 (95% CI, 5.9–NA) months in patients with G8 scores ≥ 15 (*n* = 3) and 17.0 (95% CI, 5.7–24.6) months in those with G8 scores < 15 (*n* = 14) (Figure 5). Median PFS was 9.0 (95% CI, 3.7–NA) and 6.9 (95% CI, 4.9–10.1) months, respectively.

### 3.4 Safety

AEs are summarized in Table 4. All patients experienced at least one AE (17/17, 100%). Grade ≥ 3 AEs occurred in nine patients (52.9%), whereas grade ≥ 3 treatment‐related AEs were observed in five patients (29.4%). SAEs were reported in seven patients (41.2%), including two patients (11.8%) with events considered related to study treatment. AEs led to dose reduction and treatment discontinuation in 11 (64.7%) and three (17.6%) patients, respectively. Of the three discontinuations, one patient discontinued treatment because of Grade 2 treatment‐related interstitial pneumonia; the patient recovered and subsequently started S‐1 treatment. Of the two patients who discontinued treatment because of treatment‐related peripheral sensory neuropathy, one had Grade 3 neuropathy and subsequently received gemcitabine monotherapy, whereas the other had Grade 2 neuropathy and subsequently received gemcitabine plus radiotherapy before continuing gemcitabine monotherapy.

**TABLE 4:** Summary of adverse events.

AEs	Total (*N* = 17), *n* (%)	Metastatic (*n* = 13), *n* (%)	Locally advanced (*n* = 4), *n* (%)
Any AE	17 (100)	13 (100)	4 (100)
Any treatment‐related AE	17 (100)	13 (100)	4 (100)
Grade ≥ 3 AE	9 (52.9)	7 (53.8)	2 (50.0)
Grade ≥ 3 treatment‐related AE	5 (29.4)	4 (30.8)	1 (25.0)
Any AE leading to discontinuation	3 (17.6)	3 (23.1)	0 (0)
Any treatment‐related AE leading to discontinuation	3 (17.6)	3 (23.1)	0 (0)
Dose reduction in ≥ 1 cycle total	11 (64.7)	9 (69.2)	2 (50.0)
Dose reduction (gemcitabine)	6 (35.3)	4 (30.8)	2 (50.0)
Dose reduction (nab‐paclitaxel)	11 (64.7)	9 (69.2)	2 (50.0)
Any AE leading to dose reduction	11 (64.7)	9 (69.2)	2 (50.0)
Any treatment‐related AE leading to dose reduction	10 (58.8)	9 (69.2)	1 (25.0)
Any SAE	7 (41.2)	5 (38.5)	2 (50.0)
Any treatment‐related SAE	2 (11.8)	2 (15.4)	0 (0)
AEs leading to death	0 (0)	0 (0)	0 (0)
Treatment‐related AE leading to death	0 (0)	0 (0)	0 (0)

Abbreviations: AE, adverse event; SAE, serious adverse event.

The most frequent any‐grade AEs (occurring in ≥ 50% of patients) were anemia (15/17, 88.2%), decreased white blood cell count (13/17, 76.5%), decreased neutrophil count (12/17, 70.6%), increased aspartate aminotransferase level (12/17, 70.6%), hypoalbuminemia (11/17, 64.7%), increased alanine aminotransferase level (10/17, 58.8%), alopecia (15/17, 88.2%), fatigue (13/17, 76.5%), constipation (11/17, 64.7%), anorexia (11/17, 64.7%), and peripheral sensory neuropathy (11/17, 64.7%) (Table S1). The most common grade ≥ 3 treatment‐related AEs were decreased neutrophil (3/17, 17.6%) and white blood cell (2/17, 11.8%) counts. Grade ≥ 3 anemia, fatigue, peripheral sensory neuropathy, and pain each occurred in one patient (1/17, 5.9%) (Table S2). No prophylactic granulocyte colony‐stimulating factor (G‐CSF) was administered during the study period; therapeutic G‐CSF was used in one patient for neutropenia.

## 4 Discussion

In this prospective Phase II trial, biweekly GnP showed promising antitumor activity in patients aged ≥ 75 years with unresectable PC. The observed ORR (47.1%), median OS (16.8 months), and median PFS (8.3 months) were within the ranges reported for standard GnP in pivotal trials [9, 11, 12, 13] and prospective and retrospective studies involving older patients [17, 18, 30, 31, 32, 33, 34, 35, 36, 37]. The recently published GENERATE (JCOG1611) trial reported a median OS of 17.1 months and a median PFS of 6.7 months with standard GnP in Japanese patients with metastatic or recurrent PC [12], although the eligibility age range (20–75 years) differed from that of our cohort. Prospective data specifically focusing on patients aged ≥ 75 years remain limited, although this age group accounts for a substantial proportion of patients with PC [2, 4]. Notably, real‐world data indicate poorer outcomes in this population than in younger patients. For example, Lamont et al. reported that median survival among usual‐care patients aged ≥ 75 years was 6–8 weeks shorter than that among trial participants receiving the same regimen [38]. Nevertheless, prospective and observational studies consistently indicate that GnP is feasible and effective in older adults, although treatment is frequently initiated at reduced doses to improve tolerability [30, 31, 32]. Therefore, the present study provides prospective, age‐specific data on biweekly GnP treatment in this underrepresented population. The ECOG‐ACRIN EA2186 (GIANT) trial prospectively evaluated a biweekly GnP regimen in vulnerable older adults with metastatic PC and reported a median OS of 4.7 months in the GnP arm [23]. However, the GIANT population differed substantially from ours in terms of geriatric vulnerability and disease characteristics, making direct comparison between the two studies difficult.

The outcomes observed in our cohort should therefore be interpreted in the context of patient selection. We enrolled a selected population of older adults with ECOG PS 0–1 and adequate organ function and excluded patients with factors that might compromise treatment tolerability or prognosis. Nevertheless, G8 scores varied widely (range, 6–17), indicating heterogeneity in geriatric vulnerability that was not fully captured by performance status alone. In addition, 14 of 17 patients received subsequent treatment, and baseline disease characteristics, including the relatively low frequency of liver metastases and biliary drainage, may also have influenced survival outcomes. These factors should be considered when interpreting the favorable outcomes observed in this study.

In general, the prognoses of unresectable locally advanced and metastatic PC differ, with locally advanced disease often associated with more favorable outcomes [9, 12, 13, 39]. However, in the present study, survival was shorter in the locally advanced subgroup than in the metastatic subgroup (median OS: 12.1 vs. 16.8 months; median PFS: 5.5 vs. 8.5 months). We consider this unexpected finding to be primarily attributable to the low number of locally advanced cases (*n* = 4), rendering subgroup estimates unstable and highly sensitive to individual patient outcomes. In addition, the baseline characteristics of patients suggested that those with locally advanced disease tended to have poor ECOG PS and lower G8 scores, which may also have contributed to these results. Exploratory analyses demonstrated longer survival in patients with PS 0 than in those with PS 1, whereas the interpretation of the G8‐stratified analysis was limited by the small number of patients with a G8 score of ≥ 15 (*n* = 3).

Regarding safety, both hematologic and nonhematologic toxicities remained clinically relevant and should be considered when interpreting the tolerability of the regimen. Grade ≥ 3 hematologic AEs occurred in three of 17 patients (17.6%), with neutropenia being the most frequent. Across multiple studies, including large‐scale real‐world datasets, GnP has not been consistently associated with a higher incidence of grade ≥ 3 neutropenia in older patients than in younger counterparts [18, 30, 31]. Moreover, multivariate analysis did not identify age as an independent predictor of severe neutropenia [40]. However, the lower treatment intensity often used in older populations warrants caution [18, 30, 31, 32], as such adjustments may attenuate the true effect of age on neutropenia risk. In the present study, dose reduction was required in 11 of 17 patients (64.7%), underscoring the continued need for individualized dose adjustment even with a planned biweekly schedule. Given the frequent need for dose reduction, an initially reduced dose may be considered in patients with greater geriatric vulnerability, including impaired functional or nutritional status. However, our study cannot define specific selection criteria for this approach.

Moreover, treatment‐related AEs led to treatment discontinuation in three of 17 patients (17.6%), including two discontinuations due to peripheral sensory neuropathy and one due to treatment‐related interstitial pneumonia. Across studies, any‐grade neuropathy is common following GnP administration, and reported rates of grade ≥ 3 neuropathy vary widely, ranging from 0% to 22% [16, 41, 42]. Several analyses suggest that older age, more cycles, and higher GnP exposure are associated with neuropathy risk or severity, highlighting the need for careful monitoring and early dose adjustment in older adults [43, 44]. To date, no drug has been demonstrated to prevent chemotherapy‐induced peripheral neuropathy, and limb cooling/cryocompression is currently being investigated [45, 46, 47]. A planned biweekly schedule, with less frequent nab‐paclitaxel administration, may help limit cumulative neurotoxicity, but the present single‐arm study cannot determine whether the biweekly schedule reduces neuropathy compared with either the conventional GnP schedule or toxicity‐driven modification of the conventional schedule.

Notably, most patients in our cohort (14/17, 82.4%) received subsequent systemic therapy (most commonly S‐1 or fluorouracil/levoleucovorin plus nanoliposomal irinotecan). Previous randomized trials and recent real‐world analyses have shown that effective subsequent therapy significantly improves OS in advanced PC [48, 49, 50, 51, 52]. In this context, the high proportion of patients who were able to receive subsequent therapy may have contributed to the favorable survival outcomes observed in our cohort. However, given the single‐arm design, it cannot be determined whether the biweekly GnP schedule itself facilitated the delivery of subsequent therapy through improved tolerability. An important perspective for interpreting the present study is the concept of “time toxicity,” defined as the proportion of the remaining life of a patient spent on treatment‐related activities [53]. Among patients with PC, real‐world data suggest that those receiving palliative chemotherapy spend approximately 10% of their remaining life on healthcare‐related activities [54]. Although time toxicity was not prospectively measured in this trial, the biweekly GnP schedule involves fewer planned treatment visits per cycle than the conventional schedule, which may be relevant from the perspective of reducing treatment‐related time burden. This potential benefit warrants further evaluation in comparative studies.

This study has some limitations. First, it was a single‐center, single‐arm Phase II trial with a small sample size, and enrollment was discontinued early owing to an unstable supply of nab‐paclitaxel. Eight responses were observed among 17 evaluable patients (ORR, 47.1%), which was numerically higher than the prespecified expected response rate of 30%. However, because the planned sample size was not reached, the final efficacy assessment could not be completed as planned. Therefore, the efficacy findings should be interpreted cautiously. Moreover, all participants were Japanese, which may limit the generalizability of the findings to other populations. Second, the study population was highly selected, consisting of older adults with ECOG PS 0–1 and adequate organ function. Therefore, selection bias is possible, and the results may not be applicable to more vulnerable older adults commonly encountered in clinical practice. Third, both unresectable metastatic and unresectable locally advanced disease were included, and the number of locally advanced cases was particularly small. Finally, although we used the G8 score as a geriatric screening tool, we did not perform a comprehensive geriatric assessment. These limitations should be considered when interpreting the results and highlight the need for validation in larger, multicenter studies including broader older populations.

## 5 Conclusion

In this prospective, single‐center Phase II trial, biweekly GnP administration showed promising antitumor activity in a selected population of adults aged ≥ 75 years with unresectable PC. Because enrollment ended before the planned sample size was reached and the study population was selected based on ECOG PS 0–1 and adequate organ function, the findings should be interpreted cautiously and require validation in larger studies.

## Author Contributions

**Kenji Ikezawa:** conceptualization, methodology, funding acquisition, visualization, writing – original draft, formal analysis, project administration, resources, investigation, data curation. **Toshihiro Imai:** conceptualization, investigation, writing – review and editing, methodology. **Ryoji Takada:** investigation, writing – review and editing. **Takuo Yamai:** investigation, writing – review and editing. **Nobuyasu Fukutake:** investigation, writing – review and editing. **Makiko Urabe:** investigation, writing – review and editing. **Yugo Kai:** investigation, writing – review and editing. **Kaori Mukai:** investigation, writing – review and editing. **Tasuku Nakabori:** investigation, writing – review and editing. **Kazuyoshi Ohkawa:** investigation, writing – review and editing, supervision.

## Funding

This work was supported by Osaka Foundation for the Prevention of Cancer and Cardiovascular Diseases, Adult Disease Research Grant R2.

## Ethics Statement

The study protocol was approved by the Institutional Review Board of the Osaka International Cancer Institute (approval number: 19076) and was registered in the Japan Registry of Clinical Trials (jRCTs051190038). The study was conducted in accordance with the Declaration of Helsinki and the Clinical Trials Act in Japan. Written informed consent was obtained from all participants before enrollment.

## Conflicts of Interest

Kenji Ikezawa reports lecture honoraria from Taiho Pharmaceutical, AstraZeneca, MSD, Nihon Servier, Chugai Pharmaceutical, Guardant Health Japan, Eisai, TAKATA Pharmaceutical, and Myriad Genetics. Ryoji Takada reports lecture honoraria from Taiho Pharmaceutical, Hisamitsu Pharmaceutical, Novartis, MSD, Myriad Genetics, and TEIJIN PHARMA. Takuo Yamai reports lecture honoraria from Taiho Pharmaceutical, MSD, and Bristol‐Myers Squibb Company. Kazuyoshi Ohkawa reports lecture honoraria from Chugai Pharmaceutical, Eisai, and AstraZeneca. The other authors declare no conflicts of interest.

## Supporting information


**Table S1:** Occurrence of any adverse events in the study cohort.
**Table S2:** Occurrence of Grade ≥ 3 treatment‐related adverse events in the present cohort.

## Data Availability

The data that support the findings of this study are available on request from the corresponding author. The data are not publicly available due to privacy or ethical restrictions.
