# Supplementary material for: Prospective Phase II Trial of Biweekly Gemcitabine Plus Nab‐Paclitaxel as First‐Line Therapy in Patients Aged 75 Years and Older With Unresectable Pancreatic Cancer
Source: Cancer Rep (Hoboken). 2026 Sep 14;9(9):e70691. doi: 10.1002/cnr2.70691 (PMC13574539; doi:10.1002/cnr2.70691)
Supplement: Supplementary file 1 [file CNR2-9-e70691-s001.docx]

**Supplementary Table 1. Occurrence of any adverse events in the study cohort.**

| **Any AEs** | **Total (N = 17)**  **n (%)** | | **Metastatic (n = 13)**  **n (%)** | | **Locally advanced (n = 4)**  **n (%)** | |
| --- | --- | --- | --- | --- | --- | --- |
|  | **Any Grade** | **Grade ≥3** | **Any Grade** | **Grade ≥3** | **Any Grade** | **Grade ≥3** |
| **Hematologic and laboratory events** |  |  |  |  |  |  |
| Decreased white blood cell count | 13 (76.5) | 2 (11.8) | 11 (84.6) | 2 (15.4) | 2 (50.0) | 0 (0) |
| Decreased neutrophil count | 12 (70.6) | 3 (17.6) | 10 (76.9) | 3 (23.1) | 2 (50.0) | 0 (0) |
| Anemia | 15 (88.2) | 2 (11.8) | 12 (92.3) | 2 (15.4) | 3 (75.0) | 0 (0) |
| Decreased platelet count | 5 (29.4) | 0 (0) | 5 (38.5) | 0 (0) | 0 (0) | 0 (0) |
| Increased alanine aminotransferase level | 10 (58.8) | 1 (5.9) | 8 (61.5) | 0 (0) | 2 (50.0) | 1 (25.0) |
| Increased aspartate aminotransferase level | 12 (70.6) | 1 (5.9) | 9 (69.2) | 0 (0) | 3 (75.0) | 1 (25.0) |
| Hypoalbuminemia | 11 (64.7) | 0 (0) | 9 (69.2) | 0 (0) | 2 (50.0) | 0 (0) |
| Increased creatinine level | 1 (5.9) | 0 (0) | 1 (7.7) | 0 (0) | 0 (0) | 0 (0) |
| **Non-hematologic events** |  |  |  |  |  |  |
| Constipation | 11 (64.7) | 0 (0) | 8 (61.5) | 0 (0) | 3 (75.0) | 0 (0) |
| Diarrhea | 2 (11.8) | 0 (0) | 1 (7.7) | 0 (0) | 1 (25.0) | 0 (0) |
| Anorexia | 11 (64.7) | 0 (0) | 8 (61.5) | 0 (0) | 3 (75.0) | 0 (0) |
| Nausea | 5 (29.4) | 1 (5.9) | 2 (15.4) | 0 (0) | 3 (75.0) | 1 (25.0) |
| Vomiting | 3 (17.6) | 0 (0) | 1 (7.7) | 0 (0) | 2 (50.0) | 0 (0) |
| Dysgeusia | 2 (11.8) | 0 (0) | 2 (15.4) | 0 (0) | 0 (0) | 0 (0) |
| Stomatitis | 3 (17.6) | 0 (0) | 3 (23.1) | 0 (0) | 0 (0) | 0 (0) |
| Fatigue | 13 (76.5) | 1 (5.9) | 9 (69.2) | 0 (0) | 4 (100) | 1 (25.0) |
| Peripheral sensory neuropathy^†^ | 11 (64.7) | 1 (5.9) | 8 (61.5) | 1 (7.7) | 3 (75.0) | 0 (0) |
| Alopecia | 15 (88.2) | 0 (0) | 12 (92.3) | 0 (0) | 3 (75.0) | 0 (0) |
| Rash | 2 (11.8) | 0 (0) | 1 (7.7) | 0 (0) | 1 (25.0) | 0 (0) |
| Peripheral edema | 2 (11.8) | 0 (0) | 2 (15.4) | 0 (0) | 0 (0) | 0 (0) |
| Bile duct stenosis | 3 (17.6) | 3 (17.6) | 1 (7.7) | 1 (7.7) | 2 (50.0) | 2 (50.0) |
| Interstitial pneumonia^‡^ | 1 (5.9) | 0 (0) | 1 (7.7) | 0 (0) | 0 (0) | 0 (0) |
| Lung infection | 1 (5.9) | 1 (5.9) | 1 (7.7) | 1 (7.7) | 0 (0) | 0 (0) |
| Pancreatitis | 2 (11.8) | 1 (5.9) | 0 (0) | 0 (0) | 2 (50.0) | 1 (25.0) |
| Cholecystitis | 1 (5.9) | 1 (5.9) | 1 (7.7) | 1 (7.7) | 0 (0) | 0 (0) |
| Pain | 1 (5.9) | 1 (5.9) | 1 (7.7) | 1 (7.7) | 0 (0) | 0 (0) |
| Skin infection | 1 (5.9) | 1 (5.9) | 1 (7.7) | 1 (7.7) | 0 (0) | 0 (0) |

AE, adverse event.

^†^Among the two cases of peripheral sensory neuropathy leading to treatment discontinuation, one was grade 3, and the other was grade 2; both were considered related to the study treatment.

^‡^The single case of interstitial pneumonia was grade 2 and was considered related to the study treatment.

**Supplementary Table 2. Occurrence of grade ≥3 treatment-related adverse events in the present cohort.**

| **Grade ≥3 treatment-related AEs** | **Total**  **(N = 17)**  **n (%)** | **Metastatic**  **(n = 13)**  **n (%)** | **Locally advanced**  **(n = 4)**  **n (%)** |
| --- | --- | --- | --- |
| Decreased white blood cell count | 2 (11.8) | 2 (15.4) | 0 (0) |
| Decreased neutrophil count | 3 (17.6) | 3 (23.1) | 0 (0) |
| Anemia | 1 (5.9) | 1 (7.7) | 0 (0) |
| Fatigue | 1 (5.9) | 0 (0) | 1 (25.0) |
| Peripheral sensory neuropathy | 1 (5.9) | 1 (7.7) | 0 (0) |
| Pain | 1 (5.9) | 1 (7.7) | 0 (0) |

AE, adverse event.
